# The UCSC Genome Browser database: 2021 update

**DOI:** 10.1093/nar/gkaa1070

**Published:** 2020-11-22

**Authors:** Jairo Navarro Gonzalez, Ann S Zweig, Matthew L Speir, Daniel Schmelter, Kate R Rosenbloom, Brian J Raney, Conner C Powell, Luis R Nassar, Nathan D Maulding, Christopher M Lee, Brian T Lee, Angie S Hinrichs, Alastair C Fyfe, Jason D Fernandes, Mark Diekhans, Hiram Clawson, Jonathan Casper, Anna Benet-Pagès, Galt P Barber, David Haussler, Robert M Kuhn, Maximilian Haeussler, W James Kent

**Affiliations:** Genomics Institute, University of California Santa Cruz, Santa Cruz, CA 95064, USA; Genomics Institute, University of California Santa Cruz, Santa Cruz, CA 95064, USA; Genomics Institute, University of California Santa Cruz, Santa Cruz, CA 95064, USA; Genomics Institute, University of California Santa Cruz, Santa Cruz, CA 95064, USA; Genomics Institute, University of California Santa Cruz, Santa Cruz, CA 95064, USA; Genomics Institute, University of California Santa Cruz, Santa Cruz, CA 95064, USA; Genomics Institute, University of California Santa Cruz, Santa Cruz, CA 95064, USA; Genomics Institute, University of California Santa Cruz, Santa Cruz, CA 95064, USA; Genomics Institute, University of California Santa Cruz, Santa Cruz, CA 95064, USA; Genomics Institute, University of California Santa Cruz, Santa Cruz, CA 95064, USA; Genomics Institute, University of California Santa Cruz, Santa Cruz, CA 95064, USA; Genomics Institute, University of California Santa Cruz, Santa Cruz, CA 95064, USA; Genomics Institute, University of California Santa Cruz, Santa Cruz, CA 95064, USA; Genomics Institute, University of California Santa Cruz, Santa Cruz, CA 95064, USA; Genomics Institute, University of California Santa Cruz, Santa Cruz, CA 95064, USA; Genomics Institute, University of California Santa Cruz, Santa Cruz, CA 95064, USA; Genomics Institute, University of California Santa Cruz, Santa Cruz, CA 95064, USA; Genomics Institute, University of California Santa Cruz, Santa Cruz, CA 95064, USA; Medical Genetics Center (MGZ), Munich, Germany; Genomics Institute, University of California Santa Cruz, Santa Cruz, CA 95064, USA; Genomics Institute, University of California Santa Cruz, Santa Cruz, CA 95064, USA; Howard Hughes Medical Institute, University of California Santa Cruz, Santa Cruz, CA 95064, USA; Genomics Institute, University of California Santa Cruz, Santa Cruz, CA 95064, USA; Genomics Institute, University of California Santa Cruz, Santa Cruz, CA 95064, USA; Genomics Institute, University of California Santa Cruz, Santa Cruz, CA 95064, USA

## Abstract

For more than two decades, the UCSC Genome Browser database (https://genome.ucsc.edu) has provided high-quality genomics data visualization and genome annotations to the research community. As the field of genomics grows and more data become available, new modes of display are required to accommodate new technologies. New features released this past year include a Hi-C heatmap display, a phased family trio display for VCF files, and various track visualization improvements. Striving to keep data up-to-date, new updates to gene annotations include GENCODE Genes, NCBI RefSeq Genes, and Ensembl Genes. New data tracks added for human and mouse genomes include the ENCODE registry of candidate cis-regulatory elements, promoters from the Eukaryotic Promoter Database, and NCBI RefSeq Select and Matched Annotation from NCBI and EMBL-EBI (MANE). Within weeks of learning about the outbreak of coronavirus, UCSC released a genome browser, with detailed annotation tracks, for the SARS-CoV-2 RNA reference assembly.

## INTRODUCTION

Since the debut of the UCSC Genome Browser (1) in 2001, the web-based data visualization tool has served as a digital microscope to cross-reference, interpret and analyze genome assemblies. From base pairs to contigs to chromosomes, the visualization tool allows for genome annotations to be positioned alongside the genomic DNA itself for a large number of vertebrate species and other clades of life. In this era of big data, the UCSC Genome Browser team aspires to quickly incorporate and contextualize vast amounts of genomic information.

Apart from incorporating data from researchers and consortia, the Browser also provides tools available for users to view and compare their own data with ease. Custom tracks allow users to quickly view a dataset, and track hubs allow users to extensively organize their data and share it privately using a URL. Saving a session and sharing the session URL with a colleague allows easy access to the pre-configured views of an interactive Browser image (2). Public data access also enables creators to submit their hub to our list of available ‘public hubs’ (https://genome.ucsc.edu/cgi-bin/hgHubConnect) or ‘public sessions’ (https://genome.ucsc.edu/cgi-bin/hgPublicSessions).

Accessing the underlying track data can be achieved in a variety of ways. The Table Browser (3) and RESTful API are useful to extract data from a region in many file formats such as BED or wiggle. The public MySQL server allows users to query data tables directly, and table dumps are available on the download server (https://hgdownload.soe.ucsc.edu/downloads.html) to enable bulk download and local processing of information in our database tables. Binary indexed files, liftOver files, and other large files can be found in the /gbdb/ directory hierarchy on the download server (https://hgdownload.soe.ucsc.edu/downloads.html#gbdb).

Currently, 211 genome assemblies are available on the UCSC Genome Browser, representing 107 different species. In early 2020, as a response to the urgency of supporting biomedical research for COVID-19, the SARS-CoV-2 genome assembly was released along with relevant biomedical datasets (4). With the growing number of datasets related to the RNA genome causing the pandemic, a COVID-19 landing page (https://genome.ucsc.edu/covid19.html) was created to consolidate and serve as a directory for certain information and research resources.

Given the constant production of new datasets from researchers around the world, the UCSC Genome Browser team has added support for new data types and several new display features, some of which have been suggested by the user community. New features including Hi-C, vcfPhasedTrio and bigDbSnp data visualizations are designed to assist in the interpretation of genetic variants in clinical and research settings. As always, all data and software are freely available for personal, non-profit, and academic research use.

## ANNOTATIONS AND VISUALIZATIONS

Updating existing data tracks and displaying new annotations is a key goal for the UCSC Genome Browser team as a means to better serve the genomics community. The addition of new vertebrate genome assemblies ensures that new sequences are incorporated into the Browser as consortia work to resolve gaps, repetitive regions and update chromosome assemblies.

### Improvements for clinical genetics

**Figure 1.**
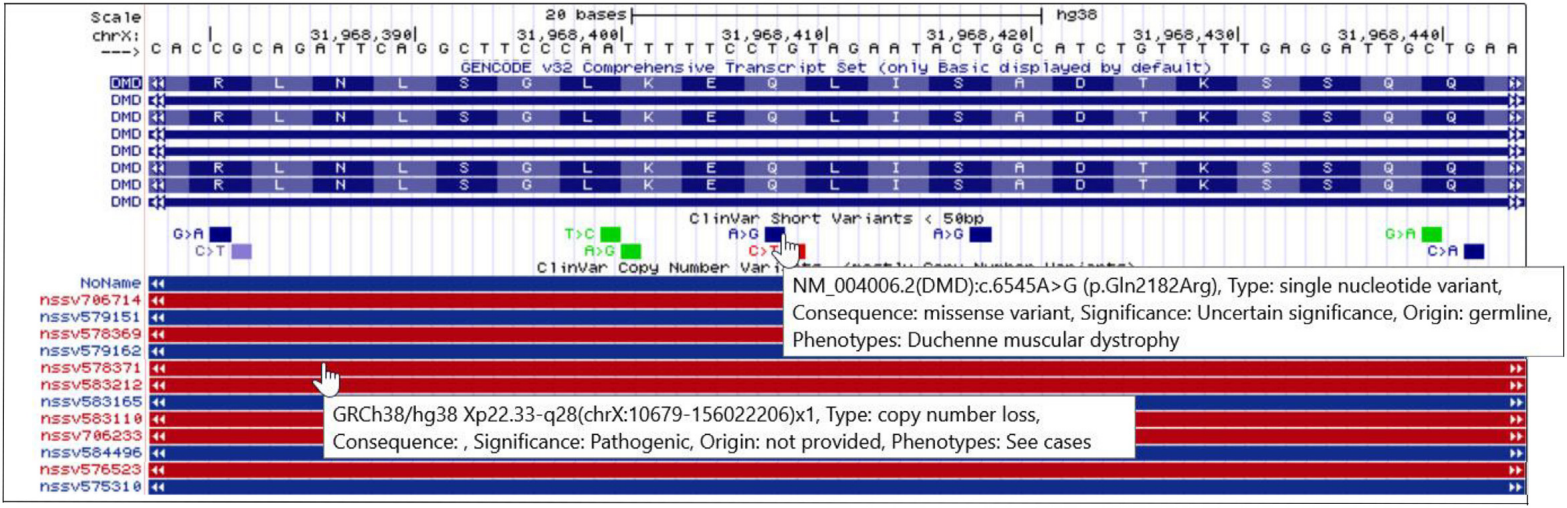
Mouse-over information is shown for the ClinVar SNVs and CNVs track. Information such as variant type, molecular consequence, clinical significance, variant origin and phenotypes are displayed.

In the past year, considerable resources were expended to upgrade the user experience for clinical variant interpretation. A primary focus of this effort was to make the detailed information readily available via mouse-overs, rather than navigating to the details page. Figure 1 shows a composite of the mouse-overs for the ClinVar Short Variants and Copy Number Variants (5), presenting the key information underlying the variant, without a click-through to the details page. On the configuration page for several tracks (ClinVar Variants, Database of Genomic Variants (6), and Leiden Open Variation Database Public Variants (7)), filters were added to allow the display of specified subsets of the data: Variant type, molecular consequence and clinical significance.

Five new tracks were created to support the assessment of sequence variants in a clinical context: gnomAD Constraint Metrics (metrics of pathogenicity per-gene and transcript regions) (Variation Group) (8); gnomAD Structural Variants (allele frequencies of SVs in the common population) (Variation Group) (8); dbVar Curated Common Structural Variants (Variation Group) (9); Automatic Variant evidence Database (AVADA) variants extracted from full-text publications (Phenotype and Literature Group) (10); the ClinGen track collection, including Gene Dosage Sensitivity (haploinsufficiency and triplosensitivity) (Phenotype and Literature Group) (11), and Problematic Regions (regions known to cause short-read sequencing analysis artifacts) (Mapping and Sequencing Group).

### Gene set updates

As new annotations are released by collaborators, the Browser team updates the corresponding tracks with the latest data. Using an automated system for many of these updates, the data are incorporated into the Browser soon after they are released at the source. Automated processes also check the data for consistency, flagging when updates indicate changes in data formats or unexpected changes in the number of records. This year, as indicated in Table 1, gene model tracks were updated for human, mouse, and other vertebrate genomes. The GENCODE Genes v32 and vM23 correspond to the default gene set, formerly named Known Gene, for human and mouse.

**Table 1.** Gene annotation tracks updated within the last year

Track name	Assembly
GENCODE Genes v33, v34	hg19, hg38
GENCODE Genes v32 (Known Gene)	hg38
GTEx V8 gene transcript expression from RNA-seq of 54 tissues	hg38
GENCODE Genes vM24, vM25	mm10
GENCODE Genes vM23 (Known Gene)	mm10
NCBI RefSeq Genes	43 assemblies
Ensembl Genes v99	43 assemblies

### New track and assembly hubs

While releasing the most useful genome annotations and assemblies to the Browser is a high priority, the sheer volume of new data exceeds our capacity to build tracks for everything. The Browser Track Hub mechanism allows users to view and share genomes and annotations without our intervention. In the past year, 17 hubs were added to the ‘public hubs’ listing, as shown in Table 2. Numerous other hubs were created and shared among colleagues, but not added to our public hub listing. A new sharing mechanism for the NCBI RefSeq assembly hubs (http://hgdownload.soe.ucsc.edu/hubs/) is now available and utilizes short links similar to the new session URLs described in the 2020 Genome Browser update (2). For example, using the RefSeq assembly accession for an elephant genome (GCF_000001905.1), a URL can be constructed such as https://genome.ucsc.edu/h/GCF_000001905.1, and will display the African savanna elephant assembly hub.

**Table 2.** New track and assembly public hubs listed by UCSC, 2019–2020

Track hub name	Provider	Assemblies
Splice Site Usage Values	Fairbrother lab: Brown University	hg19
Colocalized segments	Chen Wang & Iuliana Ionita-Laza: Kiryluk Lab, Columbia University	hg19
GENCODE COVID-19 relevant human genes	GENCODE group, European Bioinformatics Institute	hg38
NCBI dbVar	dbVar Team, NCBI	hg19, hg38
UCSC Repeat Browser	Jason Fernandes, Haussler Lab: University of California Santa Cruz	hg19, hg38
Cotney Lab Human Embryonic Heart Hub	Justin L. Cotney: University of Connecticut Health	hg19, hg38
Synonymous Constraint	Irwin Jungreis & Maxim Wolf: Broad Institute, MIT	hg19, hg38
Combined Annotation Dependent Depletion (CADD)	CADD team: University of Washington, Hudson-Alpha Institute, Berlin Institute of Health	hg19, hg38
refTSS	Takeya Kasukawa: RIKEN Center for Integrative Medical Science	hg38, mm10
TOBIAS footprint prediction	Philipp Goymann: Max Planck Institute for Heart and Lung Research	hg38,mm10
ReMap 2020 Regulatory Atlas	Benoît Ballester: ReMap human atlas	hg38, araTha1
Very Short Tandem Repeats (Polytracts)	Hui Yu: Guo Lab, University of New Mexico	hg38, mm10, rn6, dm6
DANIO-CODE Track Hub	Matthias Hörtenhuber: DANIO-CODE Data Coordination Center	danRer10, danRer11
*Wasp spider hub*	Katharina J. Hoff: University of Greifswald, Germany	*A. bruennichi*
*Xiphophorus maculatus* assembly hub	Xiphophorus Genetic Stock Center: Texas State University	*X. maculatus*
Human Global Reference Genome assembly hub	UCSC Genome Browser: Data from PanGenome Project	10 global human reference genomes
Vertebrate Genomes Project (VGP)	The Vertebrate Genomes Project	24 vertebrates
NCBI RefSeq assembly hubs	UCSC Genome Browser: Data from NCBI RefSeq	294 vertebrate assemblies

### New and updated assemblies

A total of four genome assemblies have been added to the Genome Browser within the last year; two of these are new to the Browser. In collaboration with the Monterey Bay Aquarium, the genome assembly for Gidget, a southern sea otter (enhLutNer1), was created and released. The other new genome assembly was the coronavirus, SARS-CoV-2 (wuhCor1), released as part of the effort to consolidate sequence and annotation information in one place for the virus and vaccine research communities. The assemblies for horse (equCab3), rhesus macaque (rheMac10) and gorilla (gorGor6) were updated.

### SARS-CoV-2 Genome Browser and COVID-19 annotations in Human Browsers

**Figure 2.**
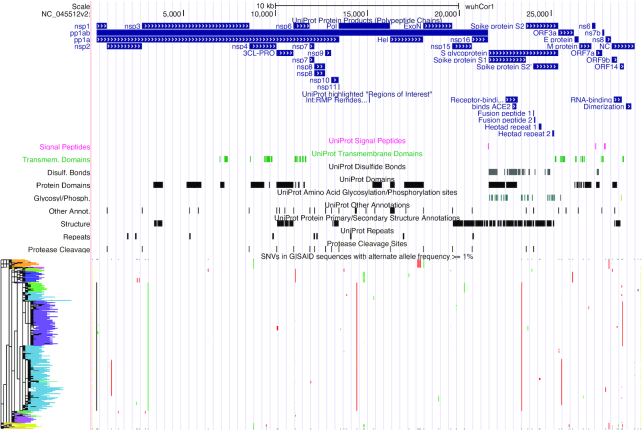
The entire viral genome in the SARS-CoV-2 browser. The default tracks shown are from the UniProt and GISAID databases. UniProt tracks shown are protein products, signal peptides, transmembrane domains, disulfide bonds, protein domains, glycosylation and phosphorylation sites, protein primary and secondary structure, cleavage sites and repeats. From GISAID, only common SNVs are displayed (alternate allele is found in at least 1% of samples), along with the phylogenetic tree showing inferred relationships between the variants.

Amidst the coronavirus pandemic, the SARS-CoV-2 assembly browser was added with datasets from major annotation databases: Protein Data Bank (12), non-coding RNA families (13), Immune Epitope Database (14), Global Initiative on Sharing All Influenza Data (GISAID) (15), and Universal Protein Resource (16). The addition of an RNA virus genome required changes to our BLAT tool to make searching feasible, and changes to the genomic display to substitute uracil for thymine. The default view and tracks for the SARS-CoV-2 genome browser are shown in Figure 2. These datasets include a wide array of information such as gene annotations, variant data, antibody epitope mappings, single-nucleotide variants (SNVs), and locally produced multiple genome alignments. Primer sets for the SARS-CoV-2 virus were added for RT-PCR, CRISPR and sequencing. We also added a Problematic Sites track with locations where masking or caution is recommended for analysis, for example, variants that are most likely sequencing artifacts and should not be used for phylogeny building (17). To accommodate the vast number of datasets being generated by researchers worldwide, crowd-sourced community annotations were added as a track. Using this mechanism, anyone can add annotations using a simple Google Sheets spreadsheet linked from the track documentation. Information about tracks released can be found in the Nature Genetics paper released in September 2020 (4).

In the human browsers (GRCh37/hg19 and GRCh38/hg38), tracks presenting meta-analysis of SARS-CoV-2 infection susceptibility and disease severity in humans were added from the COVID-19 Host Genetics Initiative (18). The new ‘lollipop’ display is used to highlight the genomic positions of SNPs that have significant effects on the phenotypes studied. The height and colors of the lollipop items represent the statistical significance along with the effect direction and size. Items can be optionally filtered by the number of studies where the SNP was identified as significant, the minimum −log_10_*P*-value and the effect size.

### Patch sequences and the mitochondrial genome (chrMT)

Last year, we began to incorporate official patch sequences from the Genome Reference Consortium into the hg38 assembly (2). This year, we added patch sequences from Genbank (19) to the hg19 assembly, and also introduced a new mitochondrial sequence, chrMT. The original hg19 genome assembly was released at UCSC along with the Genbank sequence NC_001807, designated chrM, as the mitochondrial sequence. However, the sequence preferred by the community is the revised Cambridge Reference Sequence (rCRS) NC_012920 (20). This sequence, along with many annotations, has been added to hg19 as chrMT (https://genome.ucsc.edu/cgi-bin/hgTracks?db=hg19&position=chrMT:1-16569). The original chrM sequence remains.

The patch sequences added to hg19 correspond to Genome Reference Consortium's human build 37 patch release 13 (GRCh37.p13) and can be viewed using two tracks, the Reference Assembly Fix Patch Sequence Alignments and Reference Assembly Alternate Haplotype Sequence Alignments (Mapping and Sequencing). Adding the patch sequences to the hg19 genome can cause problems for short read aligners, because some sequences appearing in alternate haplotypes now appear as repeats to aligners, when in fact they are unique in the genome, just not in the genome database. In response, an ‘analysis set’ version of the hg19 genome FASTA files (https://hgdownload.soe.ucsc.edu/goldenPath/hg19/bigZips/analysisSet/) has been added to the bigZips directory, along with indices for BWA (21,22), Bowtie2 (23), and Hisat2 (24). This analysis set is identical to NCBI’s analysis set but with UCSC style sequence names.

### ENCODE registry of candidate cis-regulatory elements (cCREs)

The ENCODE candidate cis-regulatory elements (cCREs) combined from all cell types track (Regulation Group) was added to the human (hg38) and mouse genomes (GRCm38/mm10) this year. The registry of cCREs is a core result of the integrative analysis of epigenomic and transcriptomic data sets produced from nearly two decades of ENCODE Consortium (25) efforts. cCREs are a subset of representative DNase hypersensitive sites across ENCODE and Roadmap Epigenomics (26) samples that are supported by either H3K4me3 and H3K27ac histone modifications or CTCF-binding data. A total of 926 535 elements for hg38 and 339 815 elements for mm10 were identified and classified by the ENCODE Data Analysis Center according to biochemical signatures. The cCREs track items are colored and labeled according to classification by regulatory signature. This data is prominently featured in the lead article in the July 2020 special issue of Nature marking the results from phase 3 of the ENCODE project (27).

### Eukaryotic Promoter Database (EPD) Promoters

The transcription start sites from the Eukaryotic Promoter Database (28,29) were incorporated into the Promoters from EPDnew track (Expression Group) for human (hg38 and hg19) and mouse (mm10) assemblies. These tracks represent experimentally validated promoters based on gene transcript models obtained from multiple sources (HGNC (30), GENCODE (31), Ensembl (32) and RefSeq (33)), then validated using data from CAGE (34) and RAMPAGE (35) experimental studies obtained from FANTOM 5 (36), UCSC, and ENCODE. Peak calling, clustering, and filtering based on relative expression were applied to identify the most expressed promoters and those present in the largest number of samples.

### NCBI RefSeq Select & Matched Annotation from NCBI and EMBL-EBI (MANE)

The RefSeq Select & MANE subset track (Genes and Gene Predictions Group) for the hg38 assembly is a combination of NCBI transcripts with the RefSeq Select tag, as well as transcripts with the MANE Select tag, resulting in a single representative transcript for every protein-coding gene. RefSeq Select transcripts are chosen by an NCBI pipeline to be representative of every protein-coding gene. As part of the MANE project, however, RefSeq Select transcripts that have a 100% identical match to a transcript in the Ensembl database are given the MANE Select designation. Although MANE Select is a subset of RefSeq Select, there are cases where RefSeq Select transcripts are replaced when a different MANE Select transcript is chosen. Once the MANE project is complete, RefSeq Select and MANE Select annotations should be nearly the same.

### Gene expression from GTEx project completion

The GTEx gene expression from RNA-seq track (Expression Group) for hg38 and hg19 was updated to reflect the final data release (V8) from the project. This release is based on data from 17 382 tissue samples obtained from 948 adult post-mortem individuals, reflecting a near doubling of samples and donors from the previous (V6, midpoint) release. The GTEx project final reporting is featured in the September 11 special issue of Science (37).

## SOFTWARE AND TOOL IMPROVEMENTS

Several software improvements for track visualization and overall usability of the Genome Browser have been made in the last year. The capability of the RESTful API has been expanded. We have also added new display modes for data types not previously supported, such as chromatin conformation data and phased trio haplotypes. During the year, NCBI modified the format of their dbSNP download files. This required re-engineering the pipeline for display in the Browser, which additionally provided the opportunity for improvements in functionality.

### Track visualization improvements

#### Hide empty subtracks

**Figure 3.**
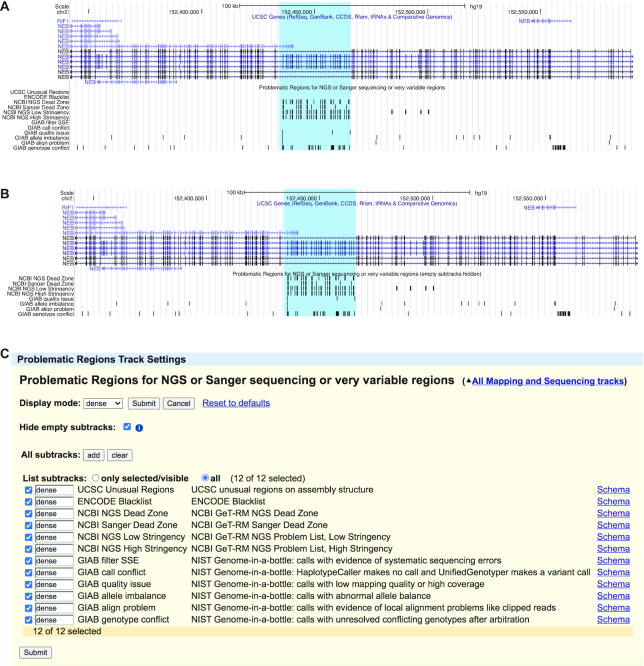
The Problematic Regions track is shown in both images. In (**A**), the original Browser display is shown for the NEB gene, a gene that has an internal duplication of some exons and short read sequencing mapping algorithms do not work well in this region. In (**B**), the ‘hide empty subtracks’ feature is used and is hiding four subtracks from the Browser display. (**C**) shows the track configuration settings used in (B).

Motivated by new large composite ENCODE track hubs (>1200 tracks of transcription factor ChIP-seq peaks), a new feature named ‘hide empty subtracks’ allows users to configure a composite to display only those subtracks containing data in the current viewing region. This feature is demonstrated in the new Problematic Regions track for the hg19 assembly, as shown in Figure 3. The track highlights regions known to cause issues in short read alignment, variant calling, or peak calling. Currently, the feature is limited to bigBed tracks inside of a multi-view composite track.

#### Collapse track items

**Figure 4.**
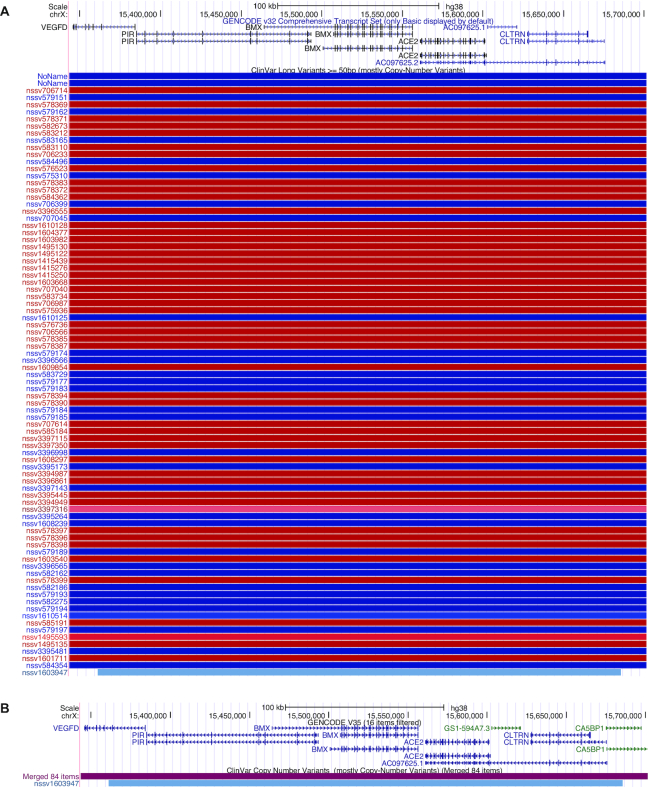

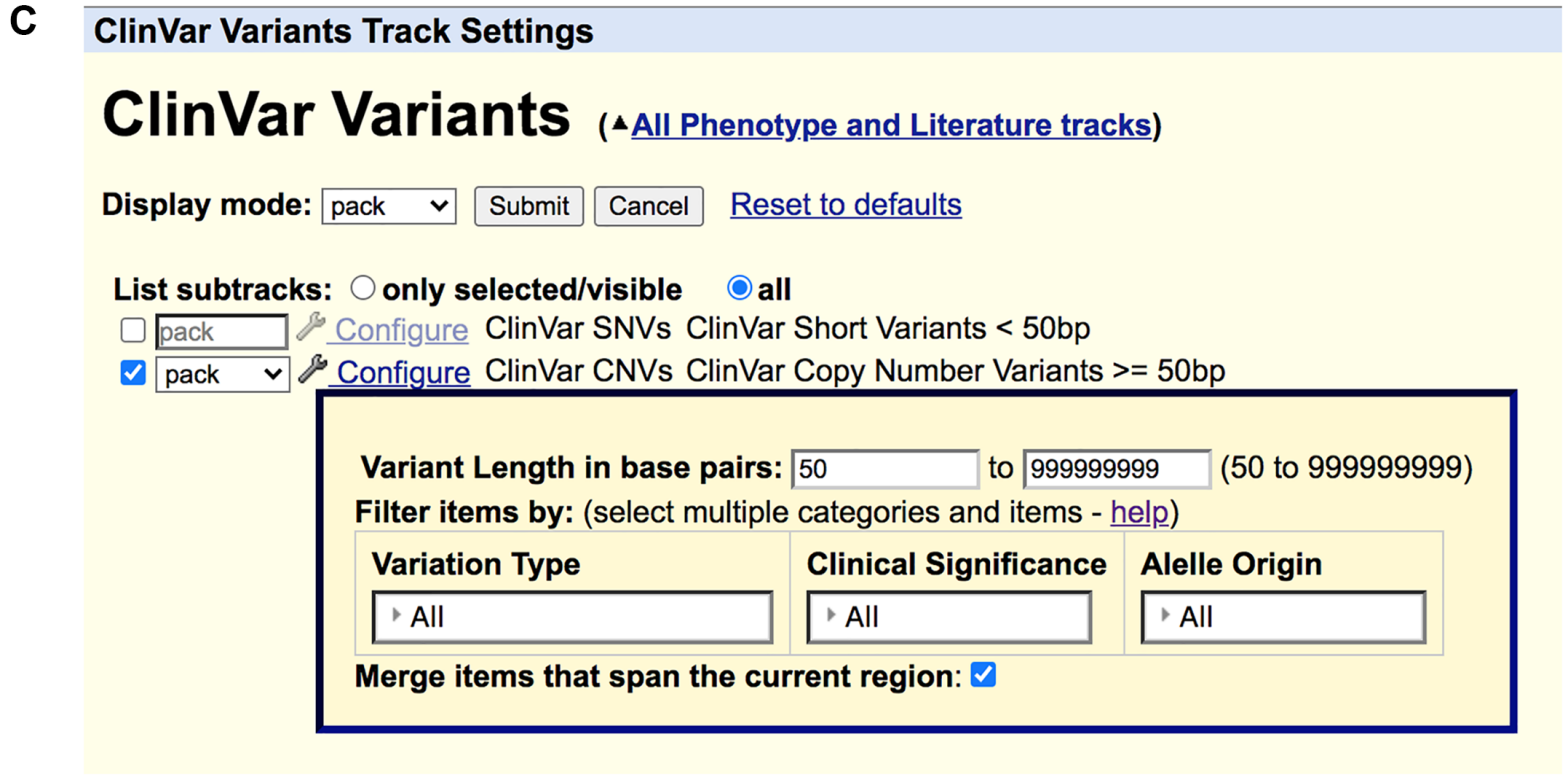
The ClinVar CNVs track is shown in both images. (**A**) The default view for the 358 090 bp region, and 85 items in the current window. (**B**) The effect of the ‘collapse track items’ feature – 84 items are no longer displayed. (**C**) The track configuration settings used in (B).

Another track visualization improvement influenced by the large amount of data available for ENCODE tracks is the ability to merge track items that span the genomic region of the viewing window. If an item neither begins nor ends within the viewing window, then this track setting suppresses display of the item. This is useful when visualizing large chromosome imbalances in tracks such as DECIPHER (38) or ClinVar CNVs (5), which have data across many megabases of DNA. A click on the merged track items restores the default view and all items are shown again. An example of collapsed track items is showcased in Figure 4 with the ClinVar CNVs track for hg38.

#### Track auto-scale changes

**Figure 5.**
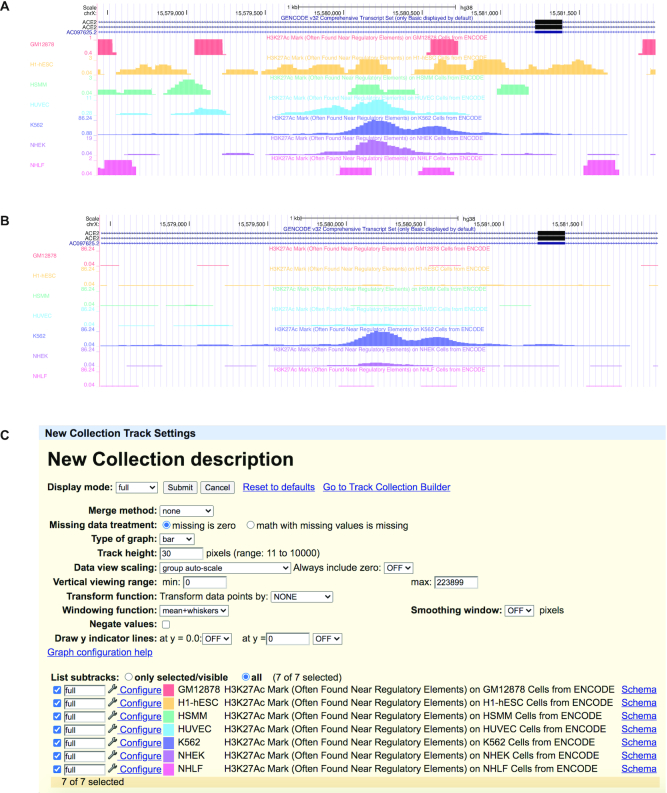
The Genome Browser figures show two auto-scale views for the same data. In (**A**), the original ‘auto-scale to data view’ setting is shown, where each track is auto-scaled to display the track's highest value. In (**B**), the new ‘group auto-scale’ setting is shown, where each track is scaled against the track with the highest value in the composite. (**C**) The track settings for the track collection in (B).

A new ‘group auto-scale’ feature is available for composite tracks made of signal subtracks in wiggle or bigWig format, such as those inside of a Track Collection. With the original auto-scale setting, which is still available, tracks within a composite track are individually scaled and change independently to different relative heights in the display. This makes it difficult to compare data in separate tracks as each track will have data somewhere in the window reaching the full height of the space. With the new ‘group auto-scale’ setting, all of the tracks in the composite track will scale to the track with the highest signal value. The new setting can be found for signal tracks within a composite group under the ‘Data view scaling:’ options. The two different auto-scale views available are shown in Figure 5.

#### RESTful API changes

The RESTful API was described in the 2020 Genome Browser update (2) and offers an easy method to extract and download annotations, chromosome lists, DNA sequences and other data from the Browser. In the original release, the tool was limited to nine track types. In the past year, support for other track types was added, including: altGraphX, barChart, chain, ctgPos, expRatio, factorSource, gvf, interact, netAlign, peptideMapping and pgSnp.

With the original release of the REST API, there were seven endpoint functions. This past year, a new endpoint function, /list/schema/, was added. This function returns the data format or track schema and configuration parameters in JSON format for a data track in a specified hub or native genome assembly.

### New track displays

#### Hi-C display

**Figure 6.**
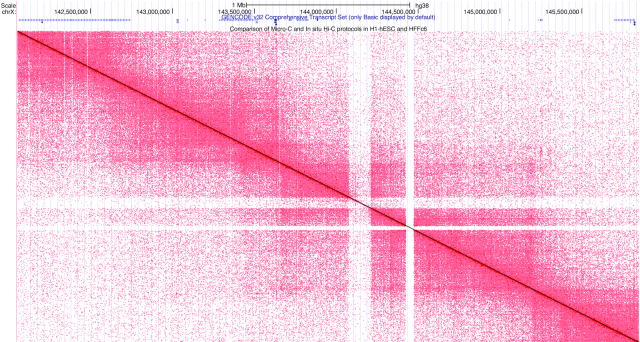
The heatmap for hg38 shows a traditional Hi-C display in ‘square’ mode (other viewing modes are ‘triangle’ and ‘arc’). Scores for this type of display correspond to how close two genomic regions are in 3-D space, with color intensity showing a high scoring interaction. The upper-left corner of the square corresponds to the left-most position of the current window, while the bottom-right corner corresponds to the right-most position of the window.

The increasing availability of chromatin conformation data, particularly since the release of the in-situ Hi-C protocol published in 2014 (39), stimulated the development of a display mode to visualize these data. Several tools already existed to view Hi-C data (JuiceBox (40), HiGlass (41), the 3D Genome Browser (42), and the WashU Epigenome Browser (43)), but there was no fully integrated solution to view the data in tandem with Genome Browser tracks or sessions. Two new Hi-C heatmap tracks are available for human (hg38 and hg19) assemblies that utilize the new display mode. Heatmaps can be configured as squares, triangles, or arcs showing interaction scores, which could indicate enhancer-promoter interactions. An example of the traditional Hi-C heatmap available on the Genome Browser is shown in Figure 6. High interaction scores indicate that more linkages were formed in the chromatin experiments and are shown with an increase in color intensity. Hi-C data from custom tracks and track hubs can also be visualized (40,44,45).

#### Personal genomics trio display

**Figure 7.**
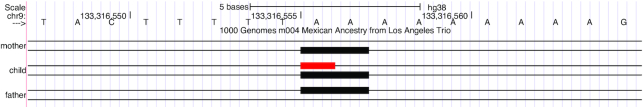
The haplotypes for the mother and father are displayed above and below the child's haplotypes. Variants predicted to be de-novo mutations in the child are shown in red.

A new track type, vcfPhasedTrio, allows for the visualization of phased personal genomics data, generally a trio consisting of a child and two parents. Two lines are drawn per sample in the underlying VCF, illustrating the haplotypes of each person's diploid genome. Variants are then drawn as tick marks on the haplotype line corresponding to which haplotype they belong, such that variants on the same line were likely inherited together. The child haplotypes are drawn in the center of each group, flanked above and below by the parent haplotypes. Haplotypes are sorted to show the transmitted alleles and ticks are colored in a variety of user-configurable settings, such as by inconsistent phasing information or predicted functional effect. The track type also allows custom sorting of the individuals, for instance, showing the child haplotypes below the parent haplotypes. The vcfPhasedTrio track type is available for both custom tracks and track hubs. The 1000 Genomes Project Family VCF trios track in hg38 utilizes this track type and is shown in Figure 7.

#### New dbSNP display and JSON format

The addition of the short genetic variants from dbSNP release b153 for the human assemblies (hg19 and hg38) introduces a new pipeline and display for the dataset. dbSNP (46) has seen exponential growth in recent releases; from roughly 324 million variants in build b150, to >700 million variants in the latest build b153. To continue providing efficient access to the data, dbSNP has redesigned its architecture and data flow. At the same time, they have made an important change to the representation of insertion/deletion variants (indels) in repetitive regions. Rather than annotating the minimal representation of the indel on the genome, which requires a choice of left-most, right-most, or arbitrary placement within the repetitive region, dbSNP now expands the reference and alternative alleles to cover the entire repetitive region on the genome.

**Figure 8.**
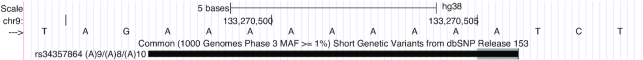
This image shows a variant in the new dbSNP b153 track inside a repetitive region. There is a deletion of one base in a range of nine identical bases, so a thin rectangle is drawn over the first eight bases to show that there is uncertain placement, and a thick rectangle is placed over the last base to show that one base is deleted from the range.

The change in dbSNP’s data format, and indel representation in particular, has led to the redesign of the dbSNP import pipeline and data representation at UCSC. A new track type, bigDbSnp, was created that uses thin and thick lines to indicate the region of uncertain placement of indels and the minimal size. An example of an arbitrarily placed variant is shown in Figure 8. The dbSNP b153 track is composed of five subtracks, four of which correspond to previously released SNP tracks (All, Common, Flagged, and Mult subsets). A new subtrack, Map Err, displays mappings with inconsistent coordinates in dbSNP download files, usually caused by difficulties in mapping certain regions between hg19 and hg38. While processing the information downloaded from dbSNP, UCSC annotates some properties of interest. These are noted on the variant's details page, and the track can be filtered to include or exclude affected variants.

The SNP tracks were previously based on related MySQL database tables, but with the release of dbSNP b153, the bigDbSnp format is a bigBed file with extra columns that contain all the necessary information to display a variant. An accompanying dbSnpDetails file includes additional data displayed on the details page for an item. With this bigDbSnp format change, the data will no longer be available as database table dumps. Instead, the bigDbSnp file for each subtrack and the shared dbSnpDetails file may be downloaded for hg19 and hg38.

## OUTREACH AND CONTACT INFORMATION

In the past year, the Genome Browser's training team provided 20 in-person seminars and workshops and three webinar-style presentations to help users take advantage of the latest features, including appearances at several national and international meetings. Due to the coronavirus pandemic, more than a dozen appearances were canceled, postponed, or converted to virtual presentations.

Outreach is supported by updates to the training documentation (https://genome.ucsc.edu/training/) with links to videos and in-depth descriptions of new Browser features. The training page also includes information on how to submit a request for a workshop and where future workshops are scheduled.

Seven new videos have been added to the Genome Browser YouTube channel (https://bit.ly/ucscVideos) in the past year. Previous videos highlighted specific problems or features of the Browser that are not obvious to the casual user or had been requested by users on our mailing lists. A new, three-part series, ‘UCSC Genome Browser Basics,’ is designed to help new users gain familiarity with the Browser and its many features. Another video, ‘UCSC Genome Browser: Coronavirus Browser SARS-CoV-2’ is designed to introduce workers in virology to the Browser and the virus. Finally, a three-part series, ‘Making Links to the UCSC Genome Browser’ is designed to help programmers, bioinformaticians, scientists using spreadsheets, and anyone wishing to share stable, customizable links to the Genome Browser.

General contact information for the UCSC Genome Browser can be found at https://genome.ucsc.edu/contacts.html, including information on accessing our email support list and an archive of previously answered mailing list questions. UCSC also maintains mirrors in Germany and Japan with the gracious assistance of Bielefeld University, Germany, and RIKEN, Japan. These sites can be found at https://genome-euro.ucsc.edu and https://genome-asia.ucsc.edu.

## FUTURE PLANS

The coming year will bring more data and tools to the UCSC Genome Browser. Additional features will be added to the phased haplotypes display. More track filtering options will be added, and the ‘hide empty subtracks’ feature will include a display of the number of tracks that are hidden in the viewing window. New features for composite tracks are in development such as introducing faceted search controls to configure complex composite tracks. Support for single-cell sequencing will continue to be developed in the coming year. We will continue to incorporate COVID-19 human annotations and SARS-CoV-2 viral genome annotations as they become available.
